# Wireless Displacement Sensing of Micromachined Spiral-Coil Actuator Using Resonant Frequency Tracking

**DOI:** 10.3390/s140712399

**Published:** 2014-07-10

**Authors:** Mohamed Sultan Mohamed Ali, Alaa AbuZaiter, Colin Schlosser, Brad Bycraft, Kenichi Takahata

**Affiliations:** 1 Faculty of Electrical Engineering, Universiti Teknologi Malaysia, Skudai, Johor 81310, Malaysia; E-Mail: ahaalaa2@live.utm.my; 2 Department of Electrical and Computer Engineering, The University of British Columbia, 2332 Main Mall, Vancouver, BC V6T 1Z4, Canada; E-Mails: colin.schlosser@gmail.com (C.S.); bbycraft@gmail.com (B.B.); takahata@ece.ubc.ca (K.T.)

**Keywords:** wireless displacement sensing, spiral-coil, micro-electro-mechanical systems, microactuators, resonant circuit

## Abstract

This paper reports a method that enables real-time displacement monitoring and control of micromachined resonant-type actuators using wireless radiofrequency (RF). The method is applied to an out-of-plane, spiral-coil microactuator based on shape-memory-alloy (SMA). The SMA spiral coil forms an inductor-capacitor resonant circuit that is excited using external RF magnetic fields to thermally actuate the coil. The actuation causes a shift in the circuit's resonance as the coil is displaced vertically, which is wirelessly monitored through an external antenna to track the displacements. Controlled actuation and displacement monitoring using the developed method is demonstrated with the microfabricated device. The device exhibits a frequency sensitivity to displacement of 10 kHz/μm or more for a full out-of-plane travel range of 466 μm and an average actuation velocity of up to 155 μm/s. The method described permits the actuator to have a self-sensing function that is passively operated, thereby eliminating the need for separate sensors and batteries on the device, thus realizing precise control while attaining a high level of miniaturization in the device.

## 1. Introduction

The displacement provided by an actuator can vary with different loads and environments. Real-time displacement measurement enables feedback control and the precise operation of such actuators, including micromachined devices, which can be used to affect microenvironments. This ability is especially relevant to biomedical devices such as active microsurgical tools and implantable drug delivery devices, in which accurate actuation is paramount. Displacement measurements for microactuators are, however, inherently challenging and often require complicated external apparatuses [1–3]. In addition, many of them require a wired interface to perform the measurement and data transfer, limiting their application range [2–4]. Some wireless techniques, including infrared sensing [5,6], laser sensing [1,7] and image processing [8,9], that do not require displacement sensors integrated with the actuators have been reported. However, these techniques require either a directed beam path or a line of sight for the measurement; therefore, they are not usable if there is any object obstructing the beam path and thus inapplicable to *in-vivo* devices. Radiofrequency (RF) sensing offers an alternative wireless method which can overcome the issues associated with these other techniques. Inductor-capacitor (*LC*) resonant circuits have been utilized in many wireless sensing applications [10–23]. Various physical parameters such as temperature [10,11], pressure [12,13], strain [14] and pH [16] have been wirelessly measured with the resonant RF approach. In these cases, *LC* circuits are arranged so that an environmental parameter causes a change in the inductance or capacitance of the circuit, leading to a shift in its resonant frequency. For displacement measurement in particular, *LC* circuits are used as a sensing element that is designed to modify its resonant frequency due to a displacement of the target actuator [24]. Often the fabrication process to directly integrate the sensing circuitry with the actuator of interest becomes complicated, especially for miniaturized devices such as micro-electro-mechanical systems (MEMS). To address this constraint, the sensing element may be fabricated as a separate element and then assembled with the target device. This approach requires a special assembly/packaging process that significantly increases the manufacturing cost. As a more sophisticated approach, actuators may be designed to have a self-sensing capability. The actuators that are capable of sensing their own displacement, using a simple detection scheme that does not require additional sensors or readout circuitry, bring various advantages, e.g., simpler device design and fabrication process, as well as ease in miniaturization, which is a critical requirement for implantable devices that must be as small as possible to minimize their medical invasiveness. To the best of the authors' knowledge, the use of this self-sensing mechanism has been limited to piezoelectric actuators [25,26] that involve complex implementations of the sensing process.

This paper reports a novel wireless displacement sensing method applicable to actuators that form *LC* resonant circuits. The proposed method is demonstrated with an out-of-plane shape-memory-alloy (SMA) spiral-coil actuator. In this actuator, the SMA coil acts as both a vertical actuator structure and a resonant circuit whose resonant frequency is displacement dependent. This monolithic configuration is aimed to reduce the overall size of the actuator while eliminating the need for sensor components and their integration with the actuator. The displacement is measured using an external sensing antenna for tracking the change in the resonant frequency toward feedback displacement control. The device design, working principle and results from the experiments performed with microfabricated devices are presented in the subsequent sections.

## 2. Device Principle and Design

**Figure 1.**
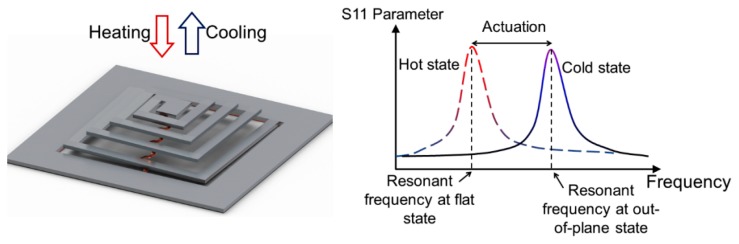
Wireless radiofrequency (RF) control and sensing of the shape-memory-alloy (SMA) spiral-coil actuator: Conceptual diagram and working principle of the device.

The spiral-coil actuator presented in this work is created by micromachining an SMA sheet (Alloy M (65 °C austenitic-phase temperature), Memry Corporation, Weil am Rhein, Germany) with a patterned SiO_2_ stress layer that is formed to produce out-of-plane displacements in the coil [27]. In its cold state, in which the SMA is in the martensite phase, the actuator is displaced vertically (out-of-plane), due to stress induced by the SiO_2_ layer patterned on the coil. As illustrated in Figure 1, the coil is actuated to a flat condition when heat is applied to the coil and the SMA enters the austenitic phase. The capacitor is built on the outer region of the SMA coil and coupled with the coil to form an *LC* circuit with a specific resonant frequency, *f*_r_, based on the inductance and capacitance values of the circuit. For the capacitor formation, the SMA substrate itself serves as one of the parallel-plate electrodes, and the other electrode is created by depositing a Cu layer with an intermediate dielectric layer, both of which are patterned on the SMA substrate to complete the capacitor. The other electrical connection between the inner end of the coil and the capacitor is made by a Cu lead that is patterned together with the Cu electrode.

**Figure 2.**
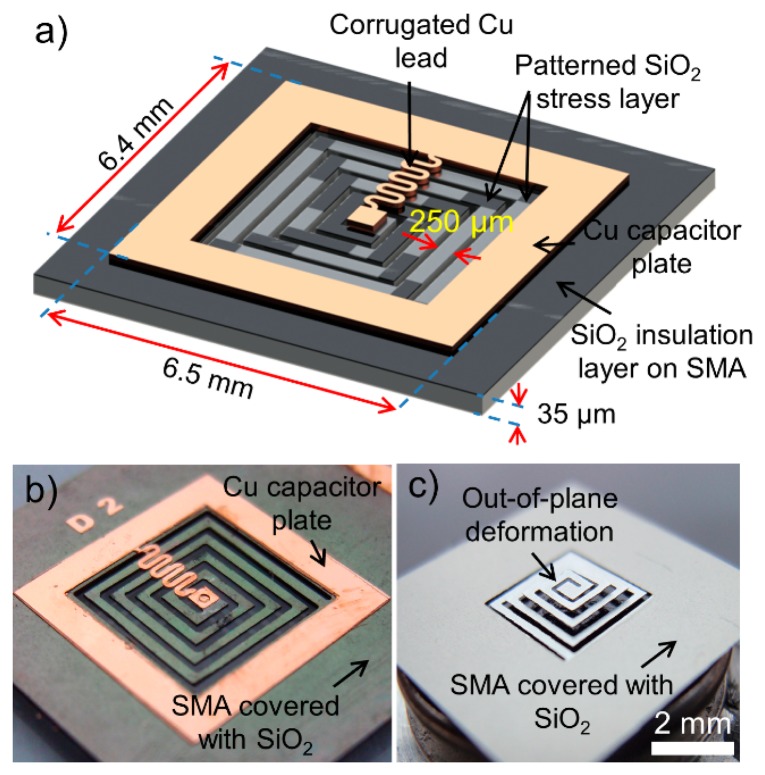
(**a**) Device design and structure (showing the backside of the device); (**b**) backside of fabricated device; (**c**) front side of fabricated device with out-of-plane deformation.

The design of the actuator is depicted in Figure 2a. The detailed actuator design and fabrication process are reported in [27]. The SMA sheet is first micromachined to form a spiral-coil before depositing a 3.5-μm-thick SiO_2_ stress layer. The SiO_2_ layer is then patterned to have a different length at each segment of the coil turns to ensure a uniform out-of-plane deformation [27]. The capacitor plate and corrugated connection to the center of the coil are formed by sputter deposition of a Cu layer followed by electroplating process to complete the out-of-plane *LC* resonant circuit fabrication. The fabricated device is shown in Figure 2b,c. Actuation of this device can be controlled wirelessly using external RF magnetic fields. When the device is exposed to the external field, an electromotive force is induced in the *LC* circuit. The electromotive force is most effectively converted to Joule heat when the field frequency, *f*_m_, matches *f*_r_; thus, by tuning *f*_m_ with respect to *f*_r_, the actuation of the SMA coil can be controlled [28,29]. The inductance of the spiral SMA coil, *L*, is dependent on the out-of-plane displacement of the coil due to the change in coil's mutual inductance caused by the variation in the gap between the coil turns [30], while the capacitance value remains constant (10 pF in this design). Consequently, since *f*_r_ is inversely proportional to the square root of *L, f*_r_ varies as the actuator displaces. For example, as depicted in Figure 1, when the actuator is at the full out-of-plane condition, *L* is the lowest level, hence the actuator has the highest *f*_r_. In contrast, when the actuator is at the complete flat condition, *L* is the highest, leading to the lowest *f*_r_. Based on these facts, it is presumed that the displacement of the actuator can be precisely determined by detecting and tracking *f*_r_, which can be performed in a wireless manner through inductive coupling between an external antenna and the inductor of the actuator. This ability enables closed-loop control of the actuation by fine tuning *f*_m_ real time with respect to the exact *f*_r_ that varies during the actuation.

## 3. Results and Discussion

In this section, the SMA spiral coil is characterized in terms of its thermal dependency of inductance, which is then extended to testing and demonstration of the wireless displacement tracking method for the out-of-plane spiral-coil SMA actuator that forms an *LC* resonant circuit.

### 3.1. Characterization of SMA Spiral Coil

**Figure 3.**
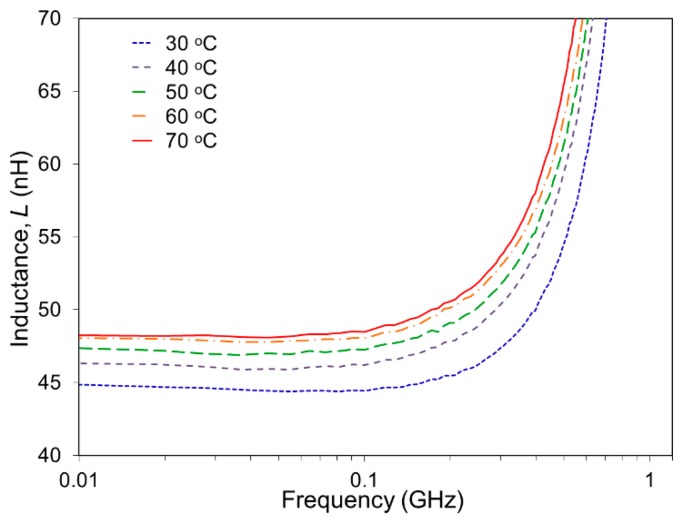
Inductance of the out-of-plane SMA spiral-coil actuator measured as a function of frequency with varying temperatures that determine the height of the coil.

The SMA spiral coil in the cold state, *i.e.*, in its full out-of-plane condition (before the capacitor formation) was first evaluated to determine the inductance value and its dependence on temperature of the coil. The spiral coil was connected to a spectrum-impedance analyzer (Agilent 4396B) using a wired interface. One wire was connected to the SMA base and the other wire to the center of the spiral coil. For a characterization purpose, the spiral coil was heated with a hot plate in this test, varying the coil's temperature from 30 °C to 70 °C stepwise with a 10 °C increment. The measurement results are plotted in Figure 3. The inductance value at 30 °C was measured to be 44.8 nH at 10 MHz with an out-of-plane height of ∼470 μm. As can be seen in the graph, the coil possessed higher inductance values at higher temperatures. When temperature was increased to 70 °C, the spiral coil archived the complete flat state, leading to the highest inductance of 48.2 nH (at the same frequency), which represents a 7.6% increase from the value at 30 °C. An important observation to note regarding the results in Figure 3 is that the rate of inductance change dropped as temperature rose. For example, the results indicate a 3.3% increase in the inductance (from 44.8 nH to 46.3 nH) when temperature increased from 30 °C to 40 °C, and this rate continued to decrease to 2.2%, 1.5%, and 0.4% for the same 10 °C rise from 40 °C, 50 °C and 60 °C, respectively. When the out-of-plane spiral coil is placed on the hot plate, heat transfer occurs naturally from the base of the spiral coil to the coil turns. In fact, the coil was observed to return to the flat geometry first from the outer turns extending to the inner turns as temperature rose because of the direction of heat flow. The outer turns of the coil produce larger changes in the inductance compared with the inner turns for a given temperature change as they have longer electrical paths, *i.e.*, larger inductance. These conditions likely caused the decreases in the rate of inductance change with temperature as described above.

### 3.2. Wireless Displacement Tracking

**Figure 4.**
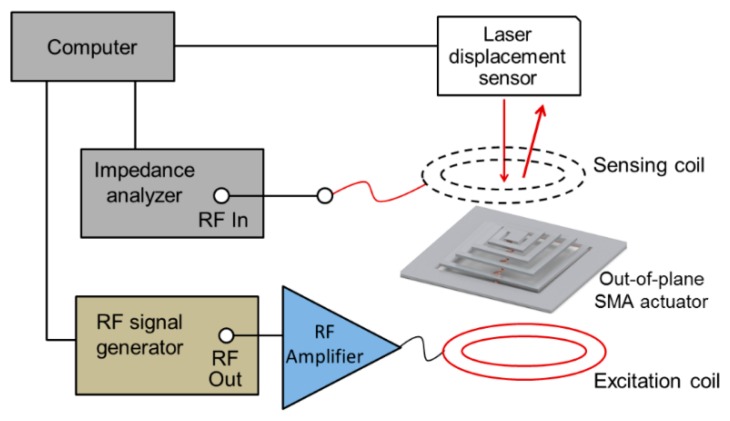
Wireless set-up used for device characterization and sensing tests.

The fabricated device with integrated capacitor was characterized in a wireless setting. The changes in actuator's *f*_r_ due to the changes in its inductance were wirelessly monitored using the set-up illustrated in Figure 4. The sensing coil/antenna (7 mm in diameter, inductance ∼380 nH) was placed 2 mm above the actuator to establish an inductive coupling with the SMA coil. As shown in Figure 4, the sensing antenna was connected to the spectrum-impedance analyzer that was interfaced with a computer through a Labview software program. The value of *f*_r_ was sampled at 20 Hz (every 50 ms) with a frequency tracking resolution of 50 kHz. The sampling frequency and resolution were restricted by computational limitations and the response time of the analyzer. The actuator was controlled by the excitation coil placed beneath the actuator for resonant heating of the SMA coil as described earlier, while the displacement at the center of the coil was precisely measured using a laser displacement sensor (LK-G32, Keyence Co., Mississauga, ON, Canada; 30-μm laser spot size, 10-nm displacement resolution).

**Figure 5.**
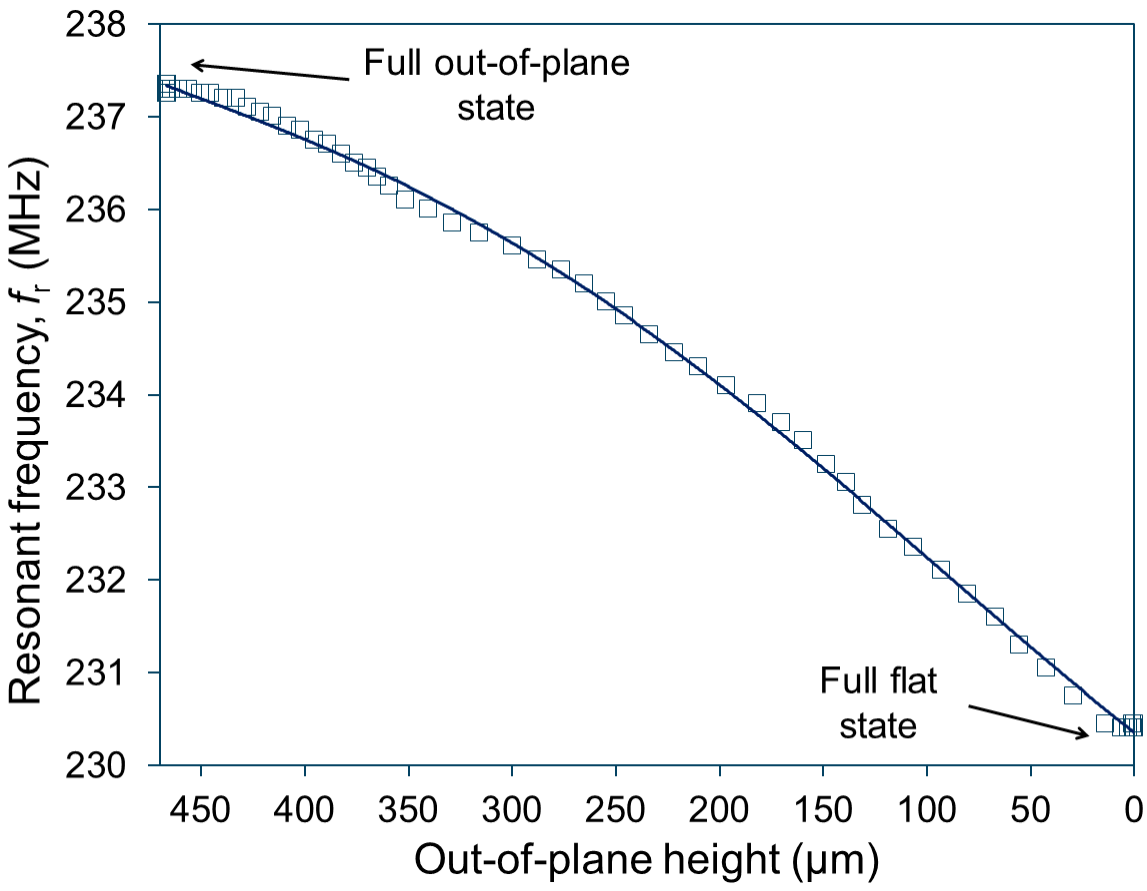
Wirelessly detected *f*_r_ *vs.* out-of-plane height of the SMA actuator varied by RF excitation at *f*_m_ = 231 MHz.

Figure 5 plots the measured relationship between *f*_r_ and the out-of-plane height when the actuator is excited at *f*_m_ of 231 MHz with an RF output power of 1 W. The result obtained shows that the actuator initially had *f*_r_ of ∼237.5 MHz at the full out-of-plane, state with the maximum actuator's height of 466 μm, and *f*_r_ reduced to ∼230.5 MHz when the actuator was operated to reach the full flat condition (*i.e.*, zero out-of-plane height in Figure 5). This plot suggests that the sensitivity of *f*_r_ is ∼15 kHz/μm on average. As also can be seen in Figure 5, *f*_r_ exhibited slightly larger changes at larger displacements (towards the flat condition). When the actuator was activated using RF fields, a higher temperature was observed at the center region of the coil, leading to the actuation of the inner turns followed by that of the outer turns [21]. (Note that this behavior is opposite to the case of the actuation test observed using the hot plate as described in the preceding subsection, in which temperature was higher at the base and outer turns of the coil.) This actuator behavior is consistent with the observed characteristic in *f*_r_ (Figure 5), *i.e.*, larger changes in *f*_r_ with larger displacements when the outer turns start to actuate.

**Figure 6.**
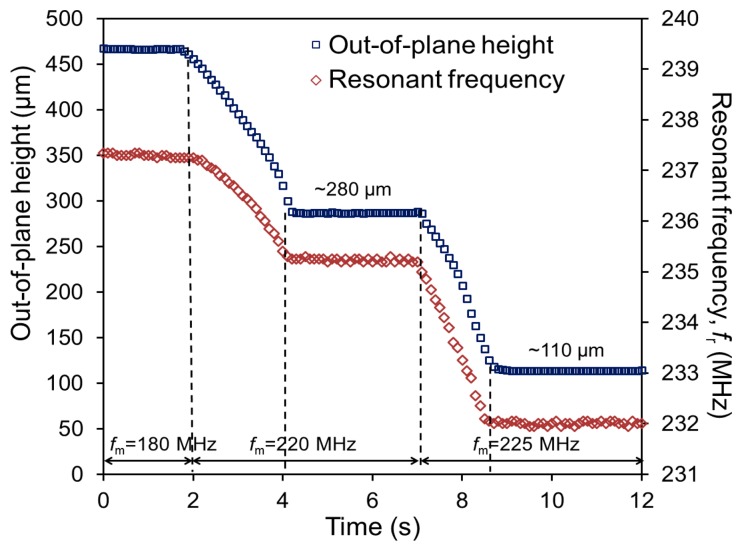
Wireless resonant tracking of *f*_r_ and measured actual displacement of the actuator operated with varying *f*_m_ (180 MHz, 220 MHz and 225MHz).

To demonstrate the ability to control the out-of-plane displacement at a desired level while monitoring it real time by tracking *f*_r_, the actuator was excited using different *f*_m_ levels at a constant output power (1 W). The results from this test are plotted in Figure 6. In this test, *f*_m_ was set at 180 MHz for the first 2 s of the measurement. During this period, almost no change was observed in *f*_r_ (237.5 MHz) due to a mismatch between *f*_m_ and *f*_r_, maintaining the initial out-of-plane height of 466 μm with nearly zero displacement. When *f*_m_ was shifted from 180 MHz to 220 MHz, the actuator was displaced from 466 μm to ∼280 μm in height. This displacement was completed in approximately 2 s, after which the actuator height was stabilized (during the time period from the 4th second to the 7th second in Figure 6). Due to this displacement, *f*_r_ shifted from 237 MHz to 235 MHz. The displacement sensitivity of *f*_r_ can be calculated to be 10.8 kHz/μm with these measurement results. As *f*_m_ was further shifted up to 225 MHz, the actuator displaced to ∼110 μm in height, causing another shift in *f*_r_ from 235 MHz to 232 MHz with an average sensitivity of 17.6 kHz/μm. The increase of the sensitivity shown in this experiment is consistent with the trend observed in Figure 5, most likely due to the same cause related to the differences between the outer and inner turns in terms of their impact on the inductance and of temperature when activated.

**Figure 7.**
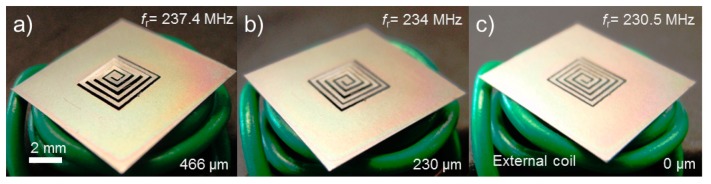
Wireless actuation of a sample device excited with *f*_m_ of (**a**) 180 MHz; (**b**) 222 MHz; (**c**) 230 MHz. Each image shows the resultant *f*_r_ (top right) and height (bottom right) of the actuator at the corresponding condition.

It is worth noting that, as can be seen in Figure 6, the second actuation exhibited a slightly higher average velocity of displacement than the first case (121 μm/s *vs.* 85 μm/s, *i.e.*, the slope of height change is larger in the second actuation). This outcome is likely a result of *f*_m_ value being closer to *f*_r_ during the second actuation compared to the first case, leading to a faster heat generation in the actuator and thus faster actuation. Figure 7 shows the actuator (still with the original SMA sheet in this particular case) at different degrees of displacement and corresponding *f*_r_ values when it was wirelessly controlled using different *f*_m_ levels as shown. These results prove that the proposed method for wireless displacement monitoring through resonance tracking is effective and feasible for the spiral-coil out-of-plane actuator. This ability is a promising path to achieving precise feedback control of the actuator. In addition, it allows one to keep tuning *f*_m_ to the value of *f*_r_ as it varies, enabling more effective RF-to-thermal power conversion. To achieve more accurate displacement tracking, the quality factor of the actuator will need to be improved. In Figure 6, it can be observed that the *f*_r_ value exhibited some fluctuation when the actuator was in the steady state, especially when the SMA's phase was closer to the austenite one (hot state). The main source of this phenomenon could be a decrease in the quality factor of the coil led by an increase in the SMA's resistivity due to a temperature rise (the resistivity of the SMA used is 82 μΩ-cm at the austenite phase whereas it is 76 μΩ-cm at the martensite phase). An improvement in the quality factor of the SMA coil, possibly by coating highly conductive metal such as Cu or Au onto the coil, is expected to increase the measurement precision and result in reduced fluctuations in the reading.

## 4. Conclusions

A novel wireless displacement sensing method for resonant-based actuators has been proposed and experimentally demonstrated with a spiral-coil SMA actuator. The SMA coil structure constituted an *LC* circuit that served as both an RF resonant heater for vertical actuation of the SMA coil and a passive sensing element for wireless displacement monitoring. The actuation was controlled using RF magnetic fields, and resultant displacements in the actuator were successfully tracked by detecting changes in the resonant frequency of the actuator itself through an external antenna. The actuation from the out-of-plane to completely flat state caused a ∼3 nH variation in the inductance of the coil, leading to a ∼7 MHz shift in its resonant frequency. The corresponding sensitivity to the out-of-plane displacement was revealed to be 10.8–17.6 kHz/μm. By being self-sensing and passively operated, the proposed actuator not only eliminates the need for additional sensors and their interface circuitry, but also does away with the challenges and constraints associated with needing to integrate power sources such as batteries for displacement sensing. These benefits potentially contribute to significant simplification and thus miniaturization of the device while increasing its longevity. The demonstrated method could be used for a variety of microactuator applications that require high-precision displacement control as well as in biomedical areas including intelligent “smart” implants where wireless interface is an essential requirement. Future work will involve the optimization of the device and the development of a closed-loop control system to achieve precise coordination and improved actuation efficiency.
